# Effects of graded dietary raw bee venom inclusion on growth, immunity, and disease resistance in Nile tilapia (*Oreochromis niloticus*)

**DOI:** 10.1038/s41598-026-69456-9

**Published:** 2026-09-08

**Authors:** Rasha M. Reda, Wafaa F. A. Ali, Doaa F. El Moghazi, Gehan K. Saleh, Mona E. Elkenawy Mansour, Eman A. Elalfy, Mohammed S. Sobh, Fatma Mahsoub

**Affiliations:** 1 Department of Aquatic Animal Medicine, Faculty of Veterinary Medicine, Zagazig University, PO Box 44511, Zagazig, Sharkia Egypt; 2 Animal Health Research Institute (AHRI) (Mansoura Branch) Agriculture Research Center (ARC), Fish disease Department, P.O. Box 246, Dokki, Giza, 12618 Egypt; 3 Animal Health Research Institute (AHRI) (Mansoura Branch) Agriculture Research Center (ARC), Biochemistry Department, P.O. Box 246, Dokki, Giza, 12618 Egypt; 4 Researcher of pharmacology, biochemistry department, Animal Health Research Institute (AHRI), Agricultural research center (ARC), Mansoura branch, P.O. Box 246, Dokki, Giza, 12618 Egypt; 5 Department of Pathology, Faculty of Veterinary Medicine, Zagazig University, PO Box 44511, Zagazig, Sharkia Egypt; 6 Department of Animal & Poultry Production, Faculty of Technology and Development, Zagazig University, PO Box 44511, Zagazig, Egypt

**Keywords:** *Aeromonas sobria*, Antioxidant status, Bee venom, Dose effect, Functional feed additives, Growth performance, Immune response, Nutraceutical, Immunology, Physiology, Zoology

## Abstract

Nowadays, studies on the application of bee venom (BV) in aquaculture have been growing due to its bioactive constituents. However, previous studies have focused on nano-formulated bee venom or parenteral administration routes on Nile tilapia (*Oreochromis niloticus*). However, the effects of graded dietary supplementation of raw venom, especially at different inclusion levels, on Nile tilapia are still limited. Therefore, this study focused on investigating the effects of bee venom supplementation at different dosage levels on growth performance, hematological and biochemical parameters, antioxidant status, immune responses, immune-related gene expression, disease resistance against *Aeromonas sobria*, and economic efficiency. One hundred and eighty Nile tilapia were randomly assigned to 4 dietary groups (CONT, BV4, BV8, and BV12) with 3 replicates for each group. The CONT group received a basal diet. The BV4, BV8, and BV12 groups administered bee venom in their diet at 4, 8, and 12 mg/kg, respectively. The growth performance was significantly improved with increasing BV inclusion (*P* ≤ 0.005), with final body weight increasing from 49.16 g in CONT to 61.96 g in BV12, while FCR decreased from 1.75 to 1.43. Hematological parameters were improved in BV8 and BV12 groups, where hemoglobin concentration increased from 9.39 g/dl in CONT to 12.28 g/dl and white blood cell count increased from 6.08 × 10^3^ / µl to 7.98 × 10^3^ / µl, respectively (*P* ≤ 0.015). Liver enzymes (ALT and AST) and stress indicators (glucose and cortisol) did not differ significantly among all the dietary groups. When the concentration of BV increased, the activities of digestive enzymes, antioxidant status, and innate immunity were significantly promoted, along with remarkable upregulation in mRNA levels of *TNF-α*, *IL-1β*, and *IL-10*. Dietary bee venom supplementation (8 and 12 mg/kg) exhibited a marked histological improvement of intestinal tissues when compared with the control group. Following the challenge with *A. sobria*, cumulative mortality progressively decreased from 53.33% in the challenged CONT group to 26.66%, 13.33%, and 6.66% in BV4, BV8, and BV12, respectively. The economic analysis showed higher profit per gram of weight gain in the BV12 and BV8 groups without increased feed cost. The BV12 group showed the highest profit per gram of weight gain (0.0839 ± 0.001) compared to the control (0.0589 ± 0.002) (*P* < 0.001). In summary, dietary supplementation with BV, especially in 12 mg/kg under the conditions of the present study, significantly improved growth, feed utilization, immune responses, and disease resistance. Therefore, 12 mg/kg can be considered the best-performing tested level of BV, and further investigations are warranted to evaluate raw bee venom as a potential functional feed additive in Nile tilapia.

## Introduction

Overall, aquaculture production has increased significantly over the past decade and continues to grow^1,2^. However, disease outbreaks within the aquaculture industry are a significant issue because of the high density at which they are cultured. A crisis like an outbreak of aquaculture disease will lead to significant economic loss and can threaten our food security^3,4^. Consequently, there is a critical need for aquaculturists to utilize new technology for the prevention and control of these diseases. This industry needs to also explore innovative feed additives to generate healthy and safe protein to ensure the sustainability of the fish aquaculture industry^5–7^. In this context, several natural dietary products, plant extracts, and beneficial microorganisms have been investigated as functional feed additives to enhance immune responses and resistance to bacterial pathogens, including Aeromonas spp., in cultured fish^8–10^.

Bee-derived products (such as propolis, royal jelly, honey, pollen, and bee venom) are being studied in depth as possible therapeutic nutraceuticals in aquaculture feed due to their wide range of bioactive properties^11,12^. Bee venom is a colorless and acidic liquid (around 88% water) secreted from the bee’s venom glands for defense against predators^13,14^. There are multiple bioactive compounds within bee venom, including, but not limited to, peptides (melittin and apamin) and enzymes (phospholipase A2 and hyaluronidase) as well as bioactive amines (histamine and dopamine) that combine to provide the venom with its remarkable biological characteristics^15–17^. As a group, the components of the venom will contribute to its antimicrobial and anti-inflammatory activity as well as contain the potential for therapeutic uses^18,19^. Bee venom contains several peptides, the major one being melittin, which can interact with biological membranes and has been associated with antimicrobial and immunomodulatory activities^15,20–25^. Phospholipase A2 and other venom components can influence inflammatory and cellular responses^16,22,24^. However, the biological effects of bee venom are concentration-dependent, and excessive exposure could lead to adverse effects; thus, it is important to determine effective ranges for inclusion in diets while taking safety into account.

Although the effects of bee venom have been studied as a feed additive for aquaculture, there are still many knowledge gaps. One study examined the toxic and therapeutic effects of bee venom administered to fingerlings of Nile tilapia by intramuscular injection^26^. Another one focused on the effect of BV compared with other feed additives (liposomal vitamin C and coenzyme Q10) added to the diet of Nile tilapia. In that study, only one level of BV was applied (4 mg/kg). This dietary level was derived from a study carried out previously in thin-lipped mullet and not Nile tilapia^12^. Another recent study aimed to use nanoparticles of loaded bee venom formulations to increase their delivery efficiency and bioavailability, but they did not test the effect of natural unmodified bee venom, which can be easier and cheaper than nano-chitosan loaded with BV^27^.

Teiba et al.^12^ studied the biological effects of raw bee venom at a 4 mg/kg diet in Nile tilapia and obtained considerable results; however, this dietary level of bee venom was chosen based on previous research done in thin-lip mullet rather than Nile tilapia. Hence, while 4 mg/kg is an acceptable starting point as it has already been tested in the target fish, the suitability of this dietary level for Nile tilapia and the potential benefits of higher inclusion levels remain to be clarified.

Furthermore, the study conducted by El Basuini et al.^26^ involved injection of bee venom in Nile tilapia in different doses (3, 6, and 9 mg/kg), leading to improved antioxidant capacity as well as immune responses without any effect on survival, growth performance, or body composition. However, repeated parenteral administration is labor-intensive and impractical under commercial aquaculture conditions, particularly when large numbers of fish are reared, whereas dietary supplementation can be incorporated into routine feeding practices and is therefore more readily applicable at the farm level. Nevertheless, these findings provide supportive evidence that Nile tilapia can respond biologically to bee venom across a range of doses and provide a rationale for exploring dietary inclusion levels above the previously tested 4 mg/kg level.

Accordingly, the present study was designed to evaluate the effects of graded dietary inclusion levels of raw, unmodified bee venom at 4, 8, and 12 mg/kg of diet on growth, physiological response, immunity, intestinal morphology, and economic outcomes.

## Materials and methods

### Ethics approval

The experiments in this study have been approved by Zagazig University, Egypt, Institutional Animal Care number ZU-IACUC/2/F/171/2025, in compliance with the rules and procedures.

### Identification of bee venom (BV) bioactive compounds by gas chromatography–mass spectrometry (GC–MS)

The bee venom used was collected from hybridized Carniolan bees (*Apis mellifera* L.) in the Bee Research Department, Plant Protection Research Institute, Agricultural Research Center, Dokki, Giza, Egypt. The venom was collected using an electrical stimulation method. Bees received a mild, non-lethal electrical stimulus and stung through electrified wires over a glass plate stretched over a glass plate, allowing the venom to be deposited on the glass surface without loss of the stinger. Each colony was stimulated for 5 min using a 12 V wet-cell battery with a pulse/timer-controlled electrical stimulation system. The deposited venom was allowed to air dry under dark ambiance conditions, after which the dried crystalline venom was carefully scraped from the glass plate. The collector plate was positioned inside or at the hive entrance/under the inner cover to minimize contamination by debris, wax, and propolis. The material was used as raw, dried bee venom without chemical purification or nano-formulation. The dried venom was stored in dark glass vials at 4 °C until use. Throughout the experiment, only one batch of dried raw bee venom was used to ensure consistency of the experimental material.

The GC-TSQ 8000 Evo mass spectrometer (Thermo Scientific, Austin, TX, USA) with a capillary column of TG-5MS (30 m × 0.25 mm × 0.25 μm film thickness) was used for the analysis of the volatile and semi-volatile compounds of bee venom. The sample was prepared by derivatization of the dried bee venom with BSTFA + TMCS (1:1, v/v) and incubation at 65 °C for 1.5 h prior to injection. The column oven was initially maintained at 60 °C and then increased at 5 °C/min to 250 °C, held for 2 min., and then increased to 300 °C at 30 °C/min. The injector temperature was set at 270 °C. The carrier gas was helium with a constant flow of 1 mL/min. An AS3000 autosampler was used to automatically inject 1 µL of the diluted derivatized sample in split mode, with a 4-min solvent delay. The EI mass spectra were recorded at 70 eV ionization energy over the m/z range 50–750 in the full-scan mode. The ion source and transfer line were maintained at 200 °C and 280 °C, respectively. Tentative identification of the detected compounds was carried out by comparing their mass spectra with those in the Wiley and NIST mass spectral libraries^23,28^.

### Experimental diets

Tables 1, revealed the formulation of the basal control diet (CONT), which was developed following NRC^29^ guidelines in meeting the nutrition needs of *O. niloticus*. The three dietary treatments consisted of the basal diet fortified with BV at levels of 4 (BV4), 8 (BV8), and 12 (BV12) mg/kg of diet. To include it in the diet, the needed quantity of dried bee venom was first dissolved in distilled water and then mixed properly with the basic diet components to make sure it was well distributed. All the feed components were blended and pelleted at a 1.5 mm diameter size, after which the samples were air-dried for 48 h at room temperature and then stored in airtight bags at 4 °C until use. The levels of inclusion in the diet were selected based on previous published evidence in Nile tilapia. The level of 4 mg/kg was based on the dietary study of Teiba et al.^12^, while 8 and 12 mg/kg were selected as higher exploratory dietary inclusion levels, informed by the biological responses reported in previous bee venom studies in Nile tilapia^26^. The upper boundary (12 mg/kg) was chosen to stay within the tested dietary range and to avoid possible adverse effects from excessive bee venom supplementation^30,31^.

**Table 1:** Formulation and composition of the basal diet (g/kg).

Ingredients	Experimental diet g/Kg	Proximate composition % on dry matter basis	
Soybean meal (45% CP)	360	Dry matter	90.81
Fish meal (72% CP)	110	Total ash	7.16
Rice bran	200	Organic mater	83.65
Wheat bran	200	Crude protein	30.02
Yellow corn	60	Ether extract	8.84
Soybean oil	15	Crude fiber	6.64
Fish oil	15	Nitrogen free extract	38.15
Vitamins mixture^1^	10	GE Kcal/g diet	4.10
Dicalcium phosphate	10	DE Kcal/g diet	3.61
Minerals mixture^2^	10		
Carboxymethyl cellulose	10		
Total	1000		

^1^Vitamin premix (per kg of premix): thiamine, 2.5 g; riboflavin, 2.5 g; pyridoxine, 2.0 g; inositol, 100.0 g; biotin, 0.3 g; pantothenic acid, 100.0 g; folic acid, 0.75 g; para-aminobenzoic acid, 2.5 g; choline, 200.0 g; nicotinic acid, 10.0 g; cyanocobalamin, 0.005 g; a-tocopherol acetate, 20.1 g; menadione, 2.0 g; retinol palmitate, 100,000 IU; cholecalciferol, 500,000 IU.

^2^Mineral premix (g/kg of premix): CaHPO_4_.2H_2_O, 727.2; MgCO_4_.7H_2_O, 127.5; KCl 50.0; NaCl, 60.0; FeC_6_H_5_O_7_.3H_2_O, 25.0; ZnCO_3_, 5.5; MnCl_2_.4H_2_O, 2.5; Cu (OAc)_2_.H_2_O, 0.785; CoCl_3_.6H_2_O, 0.477; CaIO_3_.6H_2_O, 0.295; CrCl_3_.6H_2_O, 0.128; AlCl_3_.6H_2_O, 0.54; Na2SeO_3_, 0.03.

GE: the dietary gross energy calculated based on values of 5.64, 9.44, and 4.11 Kcal GE/g for CP, EE, and NFE, respectively (Blaxter, 1989). DE: dietary digestible energy, calculated according to Anderson et al. (1991) as follows: DE (MJ/kg DM) = [(0.034 CP) + (0.022 AC) − 3.5] **w**here CP is the crude protein content and AC is the available carbohydrate (nitrogen free extract).

**Table 2:** Primers sequences, target genes for SYBR green Real-Time PCR System.

Target gene	Primers sequences	Amplicon size	GenBank number	Primer efficiency (%)	Reference
***Actin Beta*** ***(β- actin)***	TGACCTCACAGACTACCTCATG	89	KJ126772.1	**96.84**	**Standen et al. (2016)**
	TGATGTCACGCACGATTTCC				
***Interleukin 1 Beta*** ***(IL-1β)***	TGGTGACTCTCCTGGTCTGA	86	XM_005457887.3	**93.40**	
	GCACAACTTTATCGGCTTCCA				
***Tumor Necrosis Factor*** ***(TNF-α)***	CCAGAAGCACTAAAGGCGAAGA	82	AY428948.1	**91.50**	
	CCTTGGCTTTGCTGCTGATC				
***Interleukin 10*** ***(IL10)***	CTGCTAGATCAGTCCGTCGAA GCAGAACCGTGTCCAGGTAA	94	XM_003441366.2	**97.40**	

### Experimental protocol

The *O. niloticus*, weighing 19.5 ± 0.30 g, was purchased from the Fish Research Unit, Faculty of Veterinary Medicine, Zagazig University, Egypt. The fish were placed in the glass aquarium measuring 80 × 40 × 30 cm and containing 60 L of dechlorinated tap water and were allowed to acclimate for 15 days under laboratory conditions. After the acclimation period, a total of 180 *O. niloticus* were randomly assigned into four groups in three independent replicate tanks per treatment (15 fish per tank). The tank was considered the experimental unit for dietary treatment. The first group represented the control group (CONT) and was fed a baseline control diet. The 2nd (BV4), 3rd (BV8), and 4th groups (BV12) were fed diets 2, 3, and 4, respectively. Fish were fed 3% biomass of each tank, divided into three meals at 09:00, 12:00, and 15:00. At each feeding time, the allocated portion was offered gradually until apparent satiation was reached. Any remainder of the daily allowance was held for the next feeding, and the same practice was continued at the second and third meals. This feeding strategy allowed for adjustments in feed intake according to the apparent appetite of the fish, standardized the daily feed allowance based on biomass among the treatments. During the experimental duration of 60 days, the water conditions were kept stable in all tanks; the water parameters were controlled and kept within their recommended ranges as per APHA^32^(temperature 27 °C ± 1.5 °C, pH 6.5 ± 0.5, dissolved oxygen 7 ± 0.5 mg/L, ammonia 0.032 ± 0.02 mg/L, and nitrite 0.03 ± 0.05 mg/L). All aquaria were subjected to a complete water change daily using a gentle syphon to draw water through a hose located on one side of the aquarium while replacing the aquarium with dechlorinated water through a hose located on the opposite side. The process was carried out gradually and kindly to avoid disturbing the fish. This management technique was applied uniformly to all experimental tanks to maintain stable water quality and to remove any leftover feed and metabolic waste.

### Growth performance parameters

The fish were weighed every two weeks to determine the performance of their growth. Prior to each weighing, fish were anaesthetized by immersion in a benzocaine solution (100 mg/L) until they reached a surgical plane of anesthesia, as indicated by loss of equilibrium and decreased response to external stimuli. After weighing, the fish were returned to fresh aerated water for recovery before being returned to their respective tanks. The weight gain (WG, g), specific growth rate (SGR, %/day), daily feed intake (DFI), average daily gain (ADG), feed conversion ratio (FCR), and relative growth rate (RGR) were all computed^33^.

### Blood sampling, hematological, biochemical, and immunological examinations

Upon completion of the feeding trial, five fish per replicate (15 fish per treatment group) were euthanized using 100 mg/L of benzocaine solution^34^. Blood samples were collected by puncturing the caudal blood vessels.

For hematological analysis, the first portion of the blood sample was drawn using EDTA-rinsed 1-mL syringes. A hemocytometer and Natt-Herrick’s stain were used to evaluate the number of total red blood cells (RBCs)^35^. The cyanmethemoglobin method was employed to evaluate the hemoglobin concentration (Hgb)^36^. Hematocrit (Hct) was determined using the microhematocrit method^37^. Mean corpuscular volume (MCV) and mean corpuscular hemoglobin concentration (MCHC) were calculated using the standard equation^38^. White blood cells (WBCs) and differential counts were evaluated using the method proposed by Stoskopf^39^.

Blood sample portion number two was taken without the use of an anticoagulant and was subjected to a 3000-rpm centrifugation for 15 min to get the serum. Serum was obtained and used to assess biochemical parameters, antioxidants, and innate immunity.

Fish alanine aminotransferase (ALT) and aspartate aminotransferase (AST) ELISA (enzyme-linked immunosorbent assay) Kits (MyBioSource, Catalogue: MBS2800393 and MBS1601734, respectively) were used to measure the enzyme activity for serum ALT and AST following the manufacturer’s instructions. The urea and creatinine levels were measured colorimetrically according to the methods of Rosenthal^40^ and Owen et al.^41^.

The commercial colorimetric kits from Abcam were used to measure the activities of lipase (Catalogue: ab102523 at OD = 405 nm) and amylase (Catalogue: ab102524 at OD = 570 nm), following the manufacturer’s guidelines. While the activities of the protease enzyme were measured fluorometrically (Ex/Em 535/587) using commercial kits from Abcam (Catalogue: ab111750).

The commercial diagnostic kits from MyBioSource, San Diego, USA, were used to assess glucose levels (Catalogue: MBS841763) and cortisol levels (Catalogue: MBS704055) as stress markers. The assessment of antioxidant status included checking the total antioxidant capacity, TAC (Catalogue: MBS2548432); superoxide dismutase, SOD (Catalogue: MBS705758); and catalase, CAT (Catalogue: MBS705697). It also included checking lipid peroxidation such as malondialdehyde, or MDA (Catalogue: MBS007853) biomarkers. The assessment was done according to the guidelines given by the manufacturer.

Serum nitric oxide (NO) activity was determined by the Griess reagent method. The turbidimetric assay of *Micrococcus lysodeikticus* was used to evaluate lysozyme activity (LYZ). For the analysis of immunoglobulin M (IgM), fish ELISA kits from MyBioSource, San Diego (Catalogue: MBS282651), were used.

### IL‑1β and TNF‑α and IL10 gene expression

Twelve spleen samples were gathered and stored at −80 °C in RNA later solution (Ambion, Thermo Fisher Scientific). The total RNA was extracted from 100 mg of spleen tissue manually using a homogenizer, with the tissues homogenized in 1 ml of Genzol™ (Geneaid Biotech Ltd., Taiwan) without DNase. The amount of RNA was determined using a Nanodrop spectrophotometer (Q5000/Quawell, Massachusetts, USA). cDNA preparation, consisting of 1 µg of total RNA, was performed using the TOPscript™ RT DryMIX (dT18) cDNA synthesis kit (Enzynomics Co., Ltd., Daejeon, Republic of Korea). Table 2 shows the primer sequences of the housekeeping gene (*β-actin*), *IL1β*, *TNF-α*, and *IL10* genes^42^. Gene expression analysis was performed using the QuantStudio™ 1 Real-Time PCR System with SYBR Green chemistry. Melting curves were performed to confirm the amplification specificity for each gene product. The amplification efficiency of the primer pairs was assessed by standard curve assays, which ranged from 91.50% to 97.40% (Table 2). In each group three independent samples were analyzed. The relative fold changes in target gene expression were calculated using the 2^−ΔΔCT^ method^43^.

### Histopathological technique

The histopathology examination was done by fixing intestinal tissues (*n* = 15) in 10% neutral buffered formalin and processing them following routine procedures and embedding in paraffin followed by sectioning at 5 μm thickness and staining with H&E as described by Abd El-Aziz et al.^44^. Additional sections were stained with alcian blue for the visualization of mucin-secreting goblet cells. Histological slides were photographed through a Leica microscope provided with a digital camera. Histological parameters such as villus length (VL), villus width (VW), and absorption surface area (ASA) were studied using 50 well-oriented villi per fish through image analysis software. Villus length was measured from its apex to the bottom, while villus width was measured at its midpoint. Absorption surface area was computed as VL x VW^45^. The histological measurements (villus length, villus width, and absorption surface area) were performed independently by an examiner, who was blinded to the experimental groups during the histological analysis.

### Challenge test

A virulent strain of *Aeromonas sobria* that was used in this test was previously isolated from diseased *O. niloticus* at the Department of Aquatic Animal Medicine of the Faculty of Veterinary Medicine, Zagazig University. The isolate was identified at the National Research Centre (Dokki, Egypt) using the automated VITEK 2-C15 system from BioMérieux (Marcy-l’Étoile, France), confirmed to be pathogenic, and maintained as glycerol stocks at −80 °C^46^. The challenged dose was selected based on a previously determined 96-h LD_50_ of 1.5 × 10⁷ CFU/mL for the *A. sobria* isolate in *O. niloticus*, determined by the same research team in the Department of Aquatic Animal Medicine of the Faculty of Veterinary Medicine, Zagazig University^47^. In brief, healthy Nile tilapia were subjected to graded bacteria inoculations intraperitoneally (from 1.5 × 10⁴ to 1.5 × 10⁹ CFU/mL), mortality was observed within 96 h, and then LD₅₀ was estimated through Probit analysis^47^.

Following a trial of 60 days, 15 fish from each trial group (BV4, BV8, and BV12) were randomly taken for bacterial challenge assay. In addition, 15 fish from the control group were subjected to the challenge test and used as positive or negative control, as mentioned below. Before bacterial inoculation, fish were anesthetized by immersion in benzocaine solution (100 mg/L)^34^ until the loss of equilibrium and the diminished response to external stimuli. Fish were administered intraperitoneally with 0.1 mL of *A. sobria* suspension prepared at the cell concentration of 1.5 × 10^7^ CFU/mL adjusted using the McFarland standard tubes. In the control group, 15 fish were injected intraperitoneally with 0.1 mL of phosphate-buffer saline, and they were termed the negative control. The other 15 fish in the control group were injected intraperitoneally with 0.1 mL of *A. sobria* suspension at cell concentration 1.5 × 10^7^ CFU/mL, which formed a positive control. The fish were kept under the environmental conditions described in Sect. 2.4 during the challenge period. For two weeks following injection, the fish were observed for any symptoms or deaths twice daily. The samples of tissues of the liver and kidneys of the dead or sick fish were then taken for the re-isolation of bacteria.

At the end of the experimental period, all remaining fish (including survivors of the bacterial challenge test and fish not allocated to the challenge assay) were humanely euthanized with an overdose of benzocaine (250 mg/L) according to the approved animal ethics protocol. The water used during the challenge test was collected in a designated treatment tank and chemically disinfected with sodium hypochlorite. A target free-chlorine concentration of 100 ppm was maintained for 12–24 h^48^, after which residual chlorine was neutralized with sodium thiosulfate. Treated water was subsequently evaluated for free residual chlorine and effective pathogen inactivation before discharging through the institutional sewage system according to institutional biosafety and wastewater management procedures^49^.

### Economic efficacy ($)

At the end of the experimental trial, economic efficacy was calculated according to the following formula^50^:

Feed cost/g body weight gain (BWG) = FCR × cost of 1 g of diet.

Profit/g weight gain = Return/g gain (price of g) − Feed cost/g gain.

Economic efficiency = Profit/feed cost per gram of weight gain.

### Statistical data analysis

Statistical analysis was done through one-way ANOVA, with Duncan’s multiple range test for post hoc comparison. Normality and equal variance assumptions were assessed through the Shapiro-Wilk test and Levene’s test, respectively. Analysis was performed on SPSS software version 25 (IBM Corp., USA). The significance level was set at *P* < 0.05. Regarding the *A. sobria* challenge, the survival probability was evaluated using the Kaplan–Meier model, and the differences among groups were evaluated using the log-rank (Mantel–Cox) test. Statistical significance was considered at *P* < 0.05.

## Result

### Bee venom GC-MS analysis

The bee venom volatile and semi-volatile compounds and their retention time and peak area % that had been determined by GC-MS analysis were displayed in Table 3. The majority of the identified metabolites were fatty acids and their derivatives, phenolic compounds, long-chain alcohols, heterocyclic compounds, and triterpenoids. E-11-Tetradecenol trimethylsilyl ether (16.45%) was one of the major constituents, followed by trimethylsilyl (9E)-Octadecenoate (11.42%) and Palmitic acid TMS derivative (7.87%). There are other metabolites that have been detected, such as gallic acid derivatives and Indole.

**Table 3:** GC–MS chromatogram Bee Venom compounds, showing retention time (min) and area (%), and library match factor (MF).

No	Retention time (RT, min.)	Compound name	Molecular formula	Peak area%	Match factor
1	10.80	CYCLOPENT[B]INDOLE,1,2,3,4-TETRAHYDRO-1-(1 H-INDOL-2-YL)−1,3,3-TRIMETHYL-, (. +-.)-	C_22_H_22_N_2_	1.32	81.1
2	15.67	PYRIDINE	C_5_H_5_N	2.7	88.0
3	19.51	3-PYRIDINECARBONITRILE,6-[2,2-BIS(METHYLTHIO)ETHENYL]−4-(4-METHOXYPHENYL)−2-METHYL-	C_18_H_18_N_2_OS	0.74	94.5
4	19.69	2-[(2’-METHYLTHIO)−3’-PYRIDYL]−4 H-3,1-BENZOXAZIN-4-ONE	C_14_H_10_N_2_O_2_	0.83	94.0
5	25.73	(2-(3-METHOXY-4- [TRIMETHYL SILYL) OXY] PHENYL) ETHOXY) (TRIMETHYL) SILANE #	C_15_H_28_O_3_Si_2_	0.78	73.0
6	25.73	Trimethylsilyl 4-methoxy-2-(2-oxo-2- (trimethylsil yl) oxy) ethoxy) benzoate	C_16_H_26_O_6_Si_2_	0.78	65.6
7	26.01	BENZOIC ACID, 3,4,5-TRIS(TRIMETHYLSILOXY)-, TRIMETHYLSILYL ESTER	C_19_H_38_O_5_Si_4_	1.12	83.1
8	26.01	Gallic acid, 4TMS derivative	C_19_H_38_O_5_Si_4_	1.12	81.0
9	27.33	Palmitic Acid, TMS derivative	C_19_H_40_O_2_Si	7.87	90.7
10	30.27	TRIMETHYLSILYL (9E)−9-OCTADECENOATE #	C_21_H_42_O_2_Si	11.42	92.5
11	32.26	E-11-Tetradecenol, trimethylsilyl ether	C_17_H_36_OSi	16.45	86.5
12	38.25	9,12,15-OCTADECATRIENOIC ACID, 2-[(TRIMETHYLSILYL)OXY]−1-[(TRIMETHYLSILYL)OXY] ETHYL ESTER, (Z, Z, Z)	C_27_H_52_O_4_Si_2_	0.6	84.7
13	40.10	9-OCTADECENOIC ACID (Z)-	C_18_H_34_O_2_	0.86	80.2
14	40.27	cis-11-Eicosenoic acid	C_20_H_38_O_2_	2.19	81.4
15	40.24	Z-(13,14-Epoxy) tetradec-11-en-1-ol acetate	C_16_H_28_O_3_	2.19	82.0
16	40.27	Erucic acid	C_22_H_42_O_2_	2.19	81.7
17	40.56	ETHANOL, 2-(9-OCTADECENYLOXY)-, (Z)	C_20_H_40_O_2_	0.57	80.4
18	44.63	1-Heptatriacotanol	C_37_H_76_O	3.07	84.6
19	44.98	LUP-20(29)-ENE-3,28-DIOL, (3á)	C_30_H_50_O_2_	1.44	79.6

### Growth performance markers

The growth rate performance was significantly enhanced through diet BV supplementation, where the best growth was seen in the BV12 group, followed by the BV8 group in comparison with the CONT group (Table 4). The final body weight in BV4, BV8, and BV12 groups increased to 15.0%, 20.5%, and 26.0%, whereas FCR reduced to 10.9%, 15.4%, and 18.3%, respectively.

**Table 4:** Growth performance in *Oreochromis niloticus* fed on bee venom-enriched diets for 60 days.

Parameters	CONT	BV4	BV8	BV12	*P*-Value
Initial Body weight (IBW, g)	19.16 ± 0.66	19.83 ± 0.88	19.50 ± 0.57	19.50 ± 0.57	0.92
Final Body weight (FBW, g)	49.16 ± 0.441^d^	56.53 ± 0.742^c^	59.26 ± 0.371^b^	61.96 ± 0.606^a^	< 0.001
Weight gain (WG, g)	30.00 ± 0.86^d^	36.70 ± 0.20^c^	39.76 ± 0.37^b^	42.46 ± 0.57^a^	< 0.001
Specific growth rate (SGR, %)	1.57 ± 0.06^c^	1.74 ± 0.05^bc^	1.85 ± 0.04^ab^	1.92 ± 0.04^a^	0.005
Daily consumed feed (DFI)	0. 08 ± 0.002^c^	0.09 ± 0.002^b^	0.09 ± 0.001^ab^	0.10 ± 0.001^a^	0.001
Average daily gain (ADG)	0.50 ± 0.01^d^	0.61 ± 0.003^c^	0.66 ± 0.006^b^	0.70 ± 0.009^a^	< 0.001
Feed conversion ratio (FCR)	1.75 ± 0.05^a^	1.56 ± 0.04^b^	1.48 ± 0.01^bc^	1.43 ± 0.02^c^	0.001
Relative growth rate (RGR, %)	157.15 ± 9.36^c^	185.83 ± 8.90^b^	204.38 ± 7.70^ab^	218.23 ± 8.09^a^	0.005
Survival rate	100%	100%	100%	100%	N/A

The values are presented as the mean ± SE. Means within the same row carrying different superscript letters are significantly different at *p* < 0.05. CONT= group served as a control without any treatment. BV4 = group fed bee venom 4 mg/kg diet. BV8 = group fed bee venom 8 mg/kg diet. BV12 = group fed bee venom 12 mg/kg diet.

Weight gain (WG, g) = Final weight (FBW, g) − Initial weight (IBW, g).

Specific growth rate (SGR, %/day) = [(ln final weight, g − ln initial weight, g) ∕ experiment duration (days)] × 100.

Daily feed intake (DFI) = the actual total amount of consumed feed per day ∕ the total number of fish in each aquarium.

Average daily gain (ADG) = FW – IW ∕ feeding trial period in days.

Feed conversion ratio (FCR) = DFI ∕ ADG.

Relative growth rate (RGR) = (FW – IW) ∕ IW × 100.

N/A: statistical comparison was not applicable because no mortality occurred in any treatment group during the 60-day feeding trial.

### Hematological markers

The RBCs of *O. niloticus* were not affected by BV supplementation (Table 5). The highest total neutrophil count among all treatments was obtained with BV12. Fish-fed diets with 8 and 12 mg/kg BV exhibited elevated Hgb, Hct, WBCs, lymphocytes, eosinophils, and monocytes relative to the CONT group. The BV-enriched groups showed an elevation in MCV in comparison to the CONT group; conversely, MCHC results were higher in the CONT group compared to the other groups fed bee venom.

**Table 5:** Hematologic indices of *Oreochromis niloticus* following 60 days of dietary bee venom supplementation.

Parameters	CONT	BV4	BV8	BV12	*P*-Value
Red Blood Cell					
RBCs (10 ^6^ /µl)	3.22 ± 0.17	3.04 ± 0.10	3.65 ± 0.23	3.86 ± 0.45	0.20
Hgb (g/dl)	9.39 ± 0.29^b^	10.06 ± 0.30^b^	11.76 ± 0.56^a^	12.28 ± 0.28^a^	0.002
Hct (%)	24.96 ± 0.85^c^	31.30 ± 1.08^b^	34.43 ± 1.37^ab^	35.55 ± 0.72^a^	< 0.001
MCV (fl.)	87.72 ± 1.75^b^	96.26 ± 0.50^a^	94.44 ± 2.20^a^	100.99 ± 2.93^a^	0.01
MCHC (g/dl)	35.54 ± 0.16^a^	34.34 ± 0.29^b^	34.15 ± 0.31^b^	34.55 ± 0.10^b^	0.01
White Blood Cell					
WBCs (10 ^3^ /µl)	6.08 ± 0.08^c^	6.58 ± 0.53^bc^	7.24 ± 0.19^ab^	7.98 ± 0.28^a^	0.015
Neutrophil (10 ^3^ /µl)	1.82 ± 0.02^b^	1.97 ± 0.06^b^	2.07 ± 0.04^b^	2.39 ± 0.13^a^	0.004
Lymphocytes (10 ^3^ /µl)	3.65 ± 0.051^b^	3.94 ± 0.31^b^	4.34 ± 0.25^ab^	4.79 ± 0.26^a^	0.04
Eosinophil (10 ^3^ /µl)	0.18 ± 0.00^b^	0.19 ± 0.01^b^	0.21 ± 0.01^ab^	0.23 ± 0.01^a^	0.04
Monocyte (10 ^3^ /µl)	0.42 ± 0.01^b^	0.46 ± 0.03^b^	0.50 ± 0.02^ab^	0.55 ± 0.03^a^	0.04

The values are presented as the mean ± SE. Means within the same row carrying different superscript letters are significantly different at *p* < 0.05. CONT, BV4, and BV8: see footnote of Table 4. RBCs: erythrocyte count; Hgb: hemoglobin concentration; HCT: hematocrit value; MCV: Mean Corpuscular Volume; MCHC: Mean Corpuscular Hemoglobin Concentration; WBCs: white blood cell count.

### Biochemical markers

Dietary bee venom did not influence ALT, AST, and urea (*P* > 0.05), while creatinine levels were significantly higher in the BV8 and BV12 groups than in the CONT and BV4 groups (*P* = 0.012; Table 6).

**Table 6:** Liver and kidney functions of *Oreochromis niloticus* following 60 days of dietary bee venom supplementation.

Treatment	CONT		BV4	BV8	BV12			*P*-Value
Liver function								
ALT (U/L)	6.00 ± 0.10	6.31 ± 0.07		6.33 ± 0.36	6.63 ± 0.24		0.34	
AST (U/L)	21.98 ± 0.43	20.42 ± 0.45		20.25 ± 0.71	20.30 ± 0.73		0.21	
Kidney function								
Urea (mg/dl)	0.38 ± 0.02		0.38 ± 0.01	0.41 ± 0.01	0.42 ± 0.02	0.467		
Creatinine (mg/dl)	0.14 ± 0.004^b^		0.15 ± 0.006^b^	0.17 ± 0.006^a^	0.17 ± 0.007^a^	0.012		

The values are presented as the mean ± SE. Means within the same row carrying different superscript letters are significantly different at *p* < 0.05. CONT, BV4, and BV8: see footnote of Table 4. ALT: Alanine aminotransferase. AST: Aspartate transaminase.

**Fig. 1:**
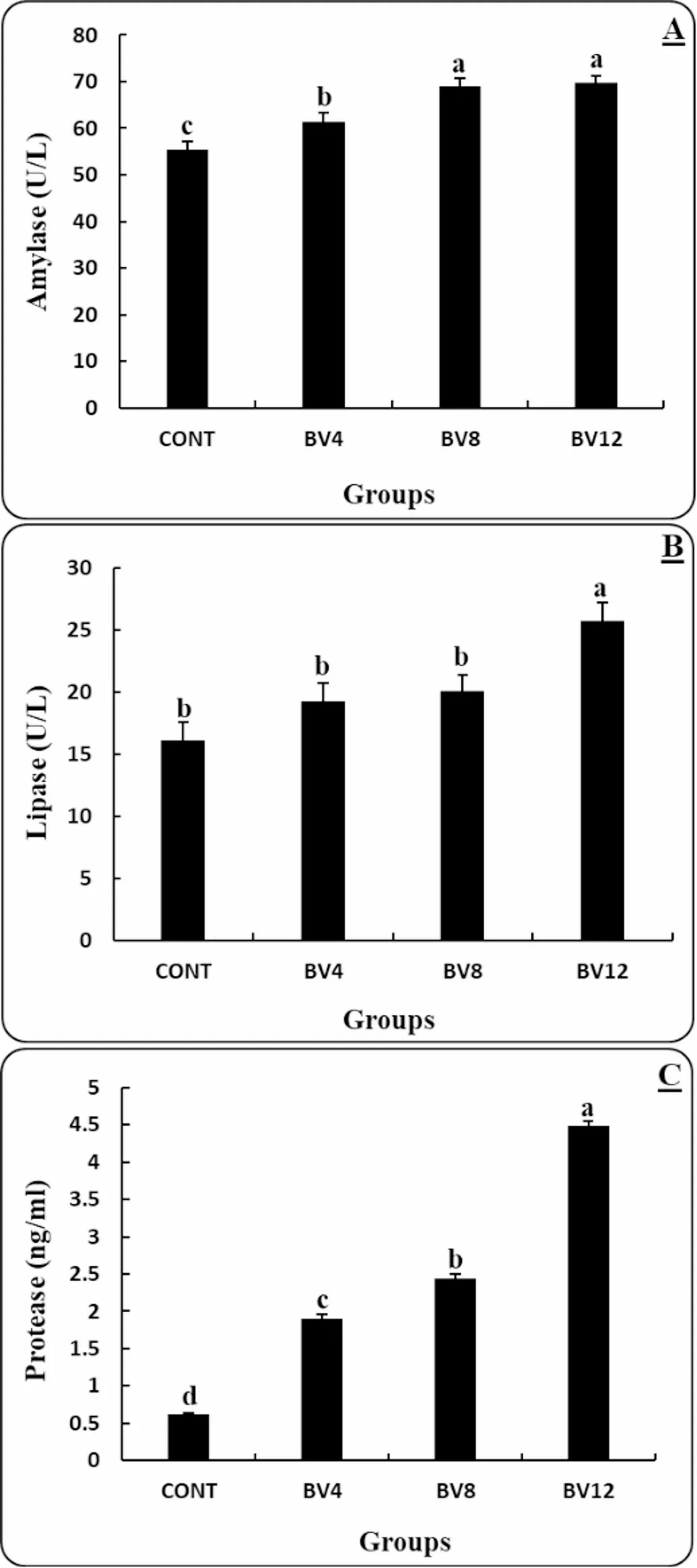
Digestive enzyme activities of *Oreochromis niloticus* following 60 days of dietary bee venom supplementation. Columns bearing different superscript letters indicate statistical differences among treatments (one-way ANOVA, *P* < 0.05). CONT, basal diet without bee venom; BV4, diet supplemented with 4 mg BV/kg; BV8, diet supplemented with 8 mg BV/kg; BV12, diet supplemented with 12 mg BV/kg.

The amylase enzyme activity attained greater values in BV12 and BV8, while BV4 showed the next highest value. The highest lipase activities were recorded at BV12 compared to the control group and other treated groups. The order of protease activities was BV12 > BV8 > BV4 > CONT (Fig. 1).

**Fig. 2:**
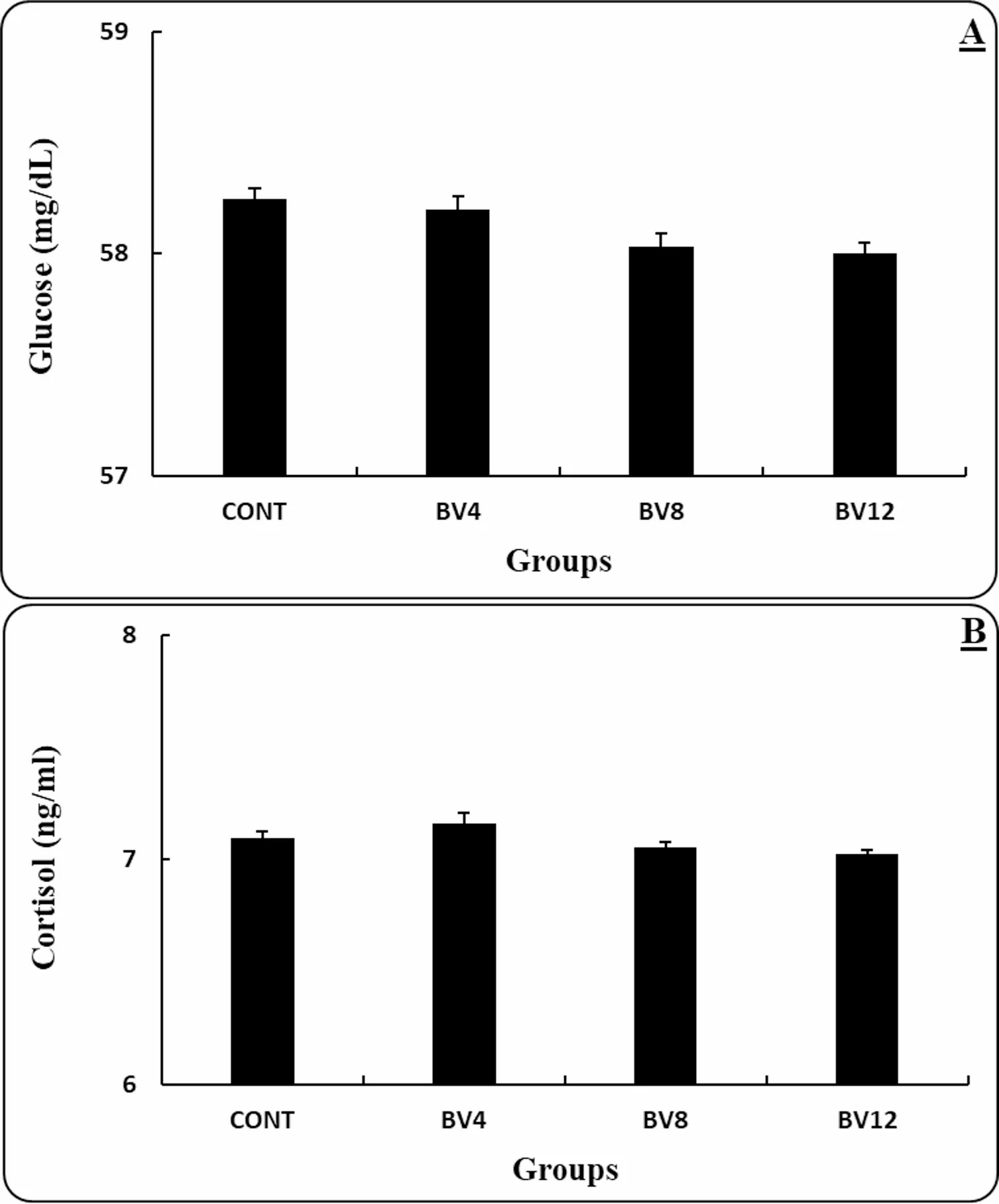
Glucose (**A**) and cortisol (**B**) levels of *Oreochromis niloticus* following 60 days of dietary bee venom supplementation. Columns bearing different superscript letters indicate statistical differences among treatments (one-way ANOVA, *P* < 0.05). CONT, basal diet without bee venom; BV4, diet supplemented with 4 mg BV/kg; BV8, diet supplemented with 8 mg BV/kg; BV12, diet supplemented with 12 mg BV/kg.

Similar values were recorded in the glucose and cortisol levels among all the experimental groups (Fig. 2).

### Oxidative stress and antioxidant markers

**Fig. 3:**
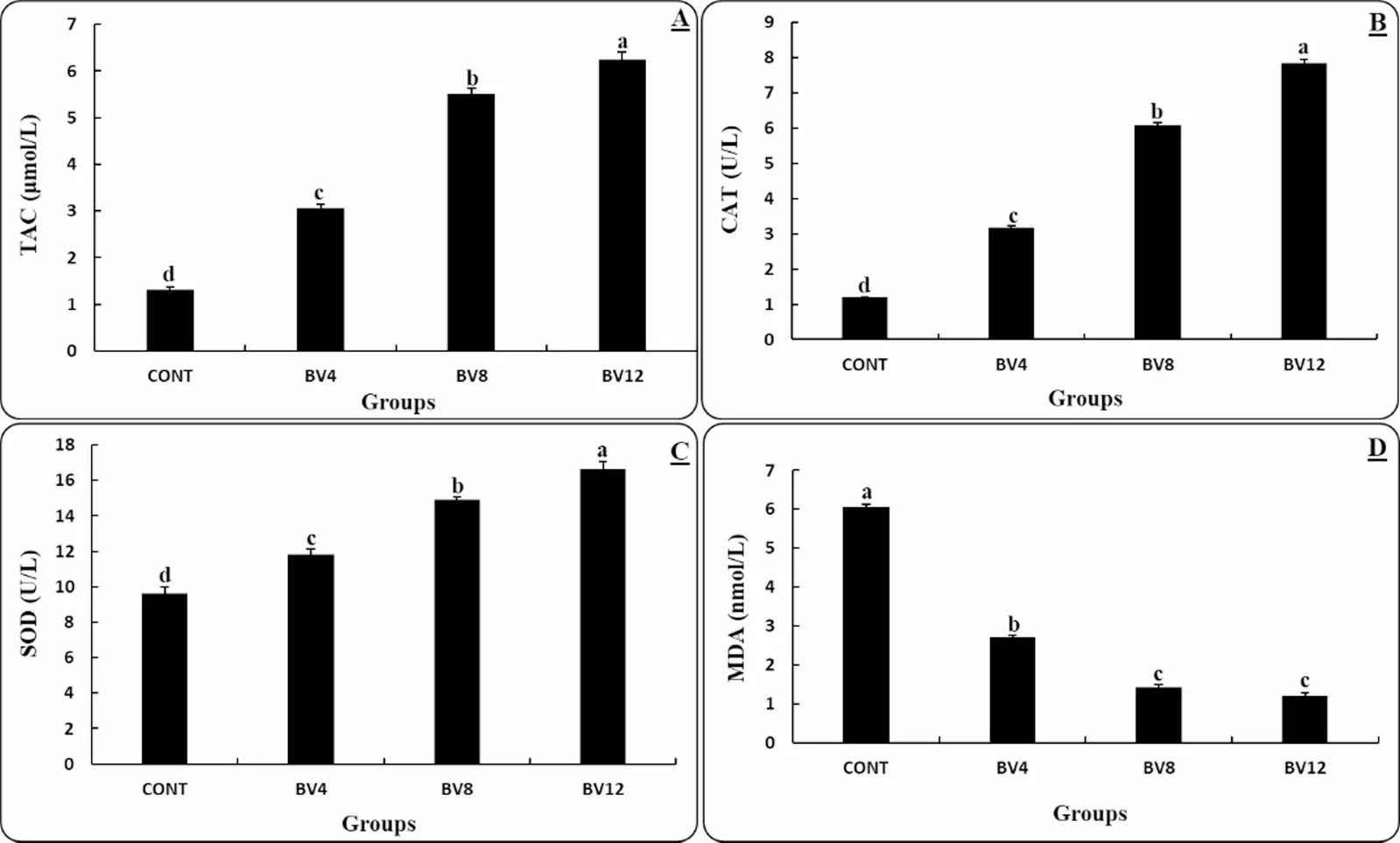
Total antioxidant capacity (TAC, **A**), catalase (CAT, **B**), superoxide dismutase (SOD, **C**) and malondialdehyde (MDA, **D**) levels of *Oreochromis niloticus* following 60 days of dietary bee venom supplementation. Columns bearing different superscript letters indicate statistical differences among treatments (one-way ANOVA, *P* < 0.05). CONT, basal diet without bee venom; BV4, diet supplemented with 4 mg BV/kg; BV8, diet supplemented with 8 mg BV/kg; BV12, diet supplemented with 12 mg BV/kg.

TAC, CAT, and SOD exhibited improvement with increasing levels of dietary BV inclusion. In contrast, the MDA levels decreased in the following order: BV8 and BV12 < BV4 < CONT (Fig. 3).

### IL‑1β, TNF‑α, and IL10 gene expression markers

**Fig. 4:**
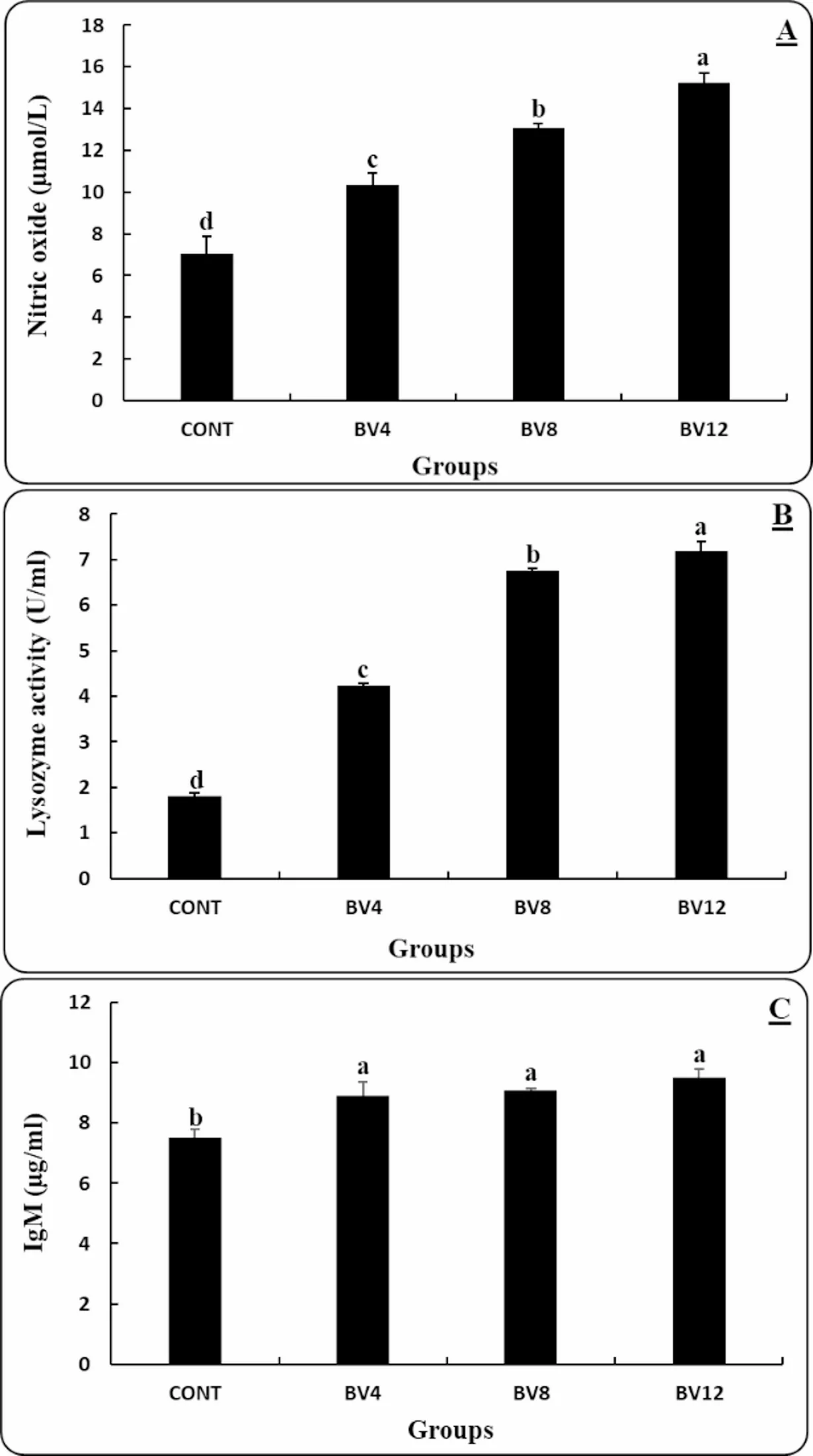
Nitric oxide (**A**), lysozyme activity (**B**) and immunoglobulin M (IgM) levels (**C**) of *Oreochromis niloticus* following 60 days of dietary bee venom supplementation. Columns bearing different superscript letters indicate statistical differences among treatments (one-way ANOVA, *P* < 0.05). CONT, basal diet without bee venom; BV4, diet supplemented with 4 mg BV/kg; BV8, diet supplemented with 8 mg BV/kg; BV12, diet supplemented with 12 mg BV/kg.

**Fig. 5:**
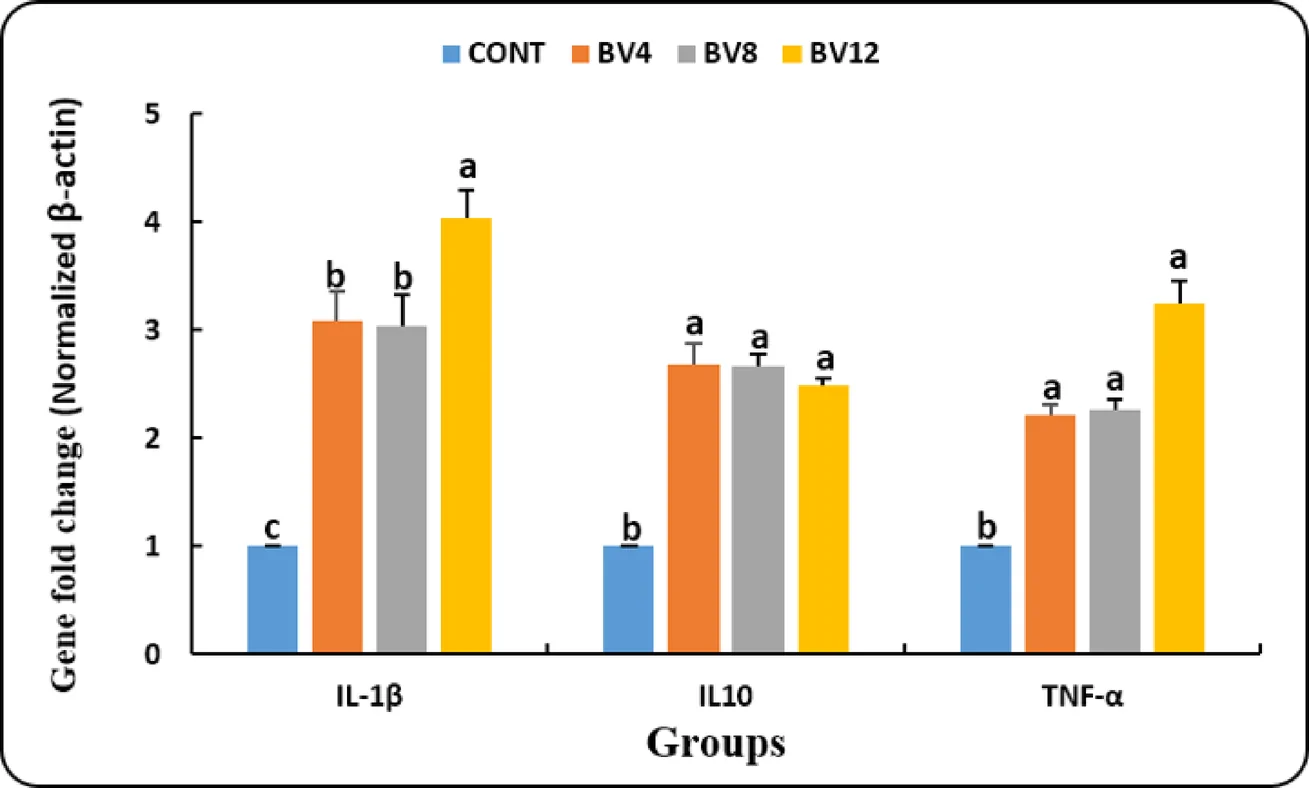
Interleukin-1β (IL-1β) (**A**), interleukin-10 (IL10) (**B**), and tumor necrosis factor-α (TNF-α) (**C**) gene **e**xpression of *Oreochromis niloticus* following 60 days of dietary bee venom supplementation. Columns bearing different superscript letters indicate statistical differences among treatments (one-way ANOVA, *P* < 0.05). CONT, basal diet without bee venom; BV4, diet supplemented with 4 mg BV/kg; BV8, diet supplemented with 8 mg BV/kg; BV12, diet supplemented with 12 mg BV/kg.

The nitric oxide, lysozyme activities, and *IL-1β* levels progressively elevated with increasing BV inclusion levels compared to the CONT group. All BV-enriched groups showed comparable levels of IgM, *IL10*, and *TNF-α*, whereas all showed higher values than CONT (Figs. 4 and 5). *IL-1β* expression increased by 3.08-, 3.04-, and 4.04-fold in the BV4, BV8, and BV12 groups, respectively. *IL10* expression increased by 2.68-, 2.66-, and 2.48-fold, respectively, whereas *TNF-α* expression increased by 2.21-, 2.26-, and 3.25-fold, respectively, compared with the CONT group.

### Histomorphological findings

**Fig. 6:**
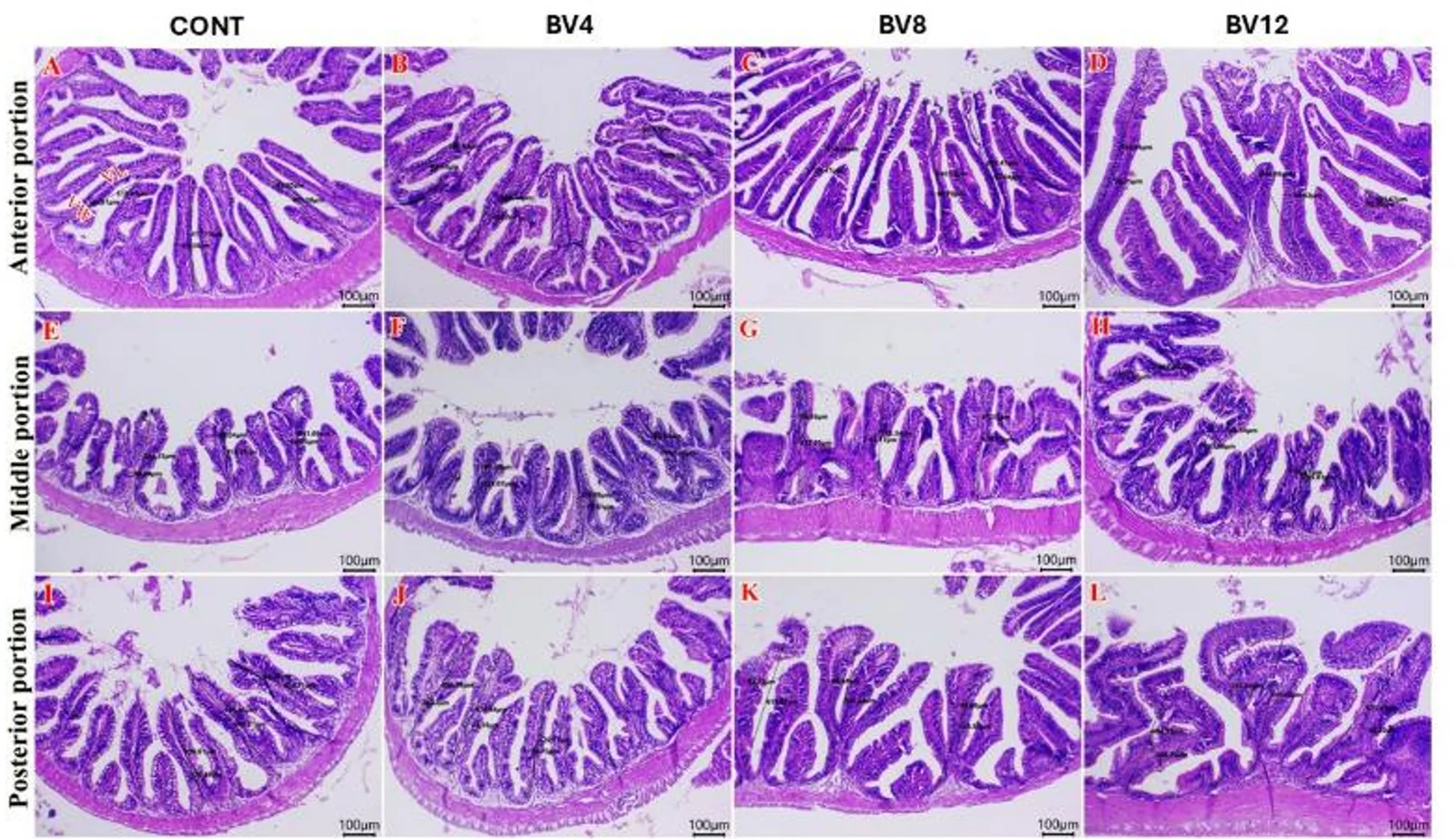
Photomicrographs of hematoxylin and eosin (H&E)-stained sections from anterior portion (**A-D**), Middle portion (**E-H**), Posterior portion (**I-L**) showing normal histological structures of columnar epithelial lining mucosal villi interspersed with goblet cells, lamina propria, submucosal layer and muscular layer. well improved intestinal parameters (villus length “VL”, villus width “VW”, absorption surface area “ASA” and number of goblet cells/villi) at intestinal portions particularly at BV8, BV12. Partially improved intestinal parameters at BV4 when compared with control group (Scale bar 100 μm). CONT, basal diet without bee venom; BV4, diet supplemented with 4 mg BV/kg; BV8, diet supplemented with 8 mg BV/kg; BV12, diet supplemented with 12 mg BV/kg.

**Fig. 7:**
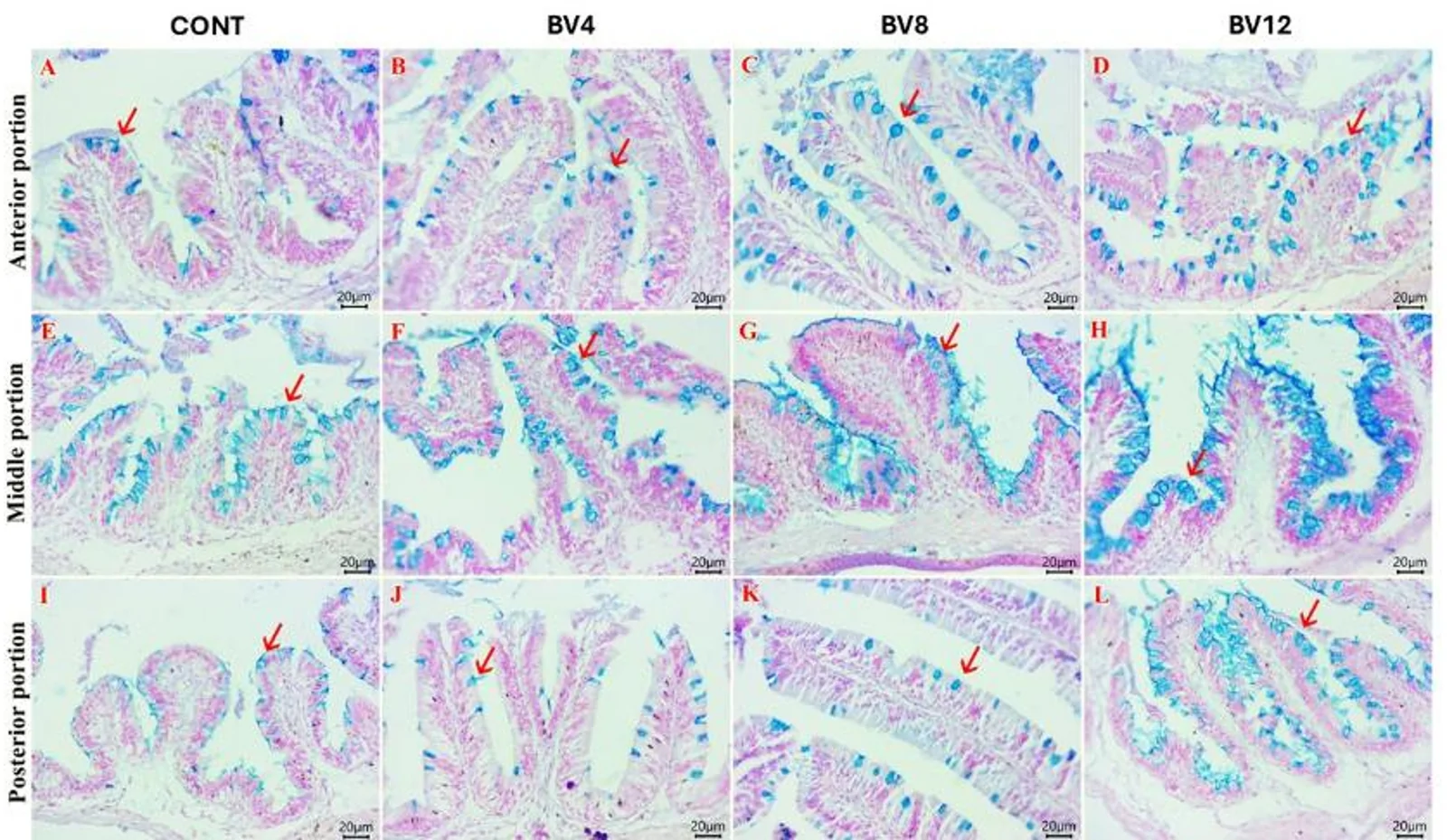
Photomicrograph of Alcian blue stained sections from anterior portion (**A-D**), Middle portion (**E-H**), Posterior portion (**I-**L) showing gradually increase in numbers of Alcian blue-stained goblet cells (arrows) from control group to BV8 with the greatest density observed in BV12 at all different portions of intestine. More goblet cells/villi at middle portions of intestine in comparison with other intestinal portions (Scale bar 20 μm). CONT, basal diet without bee venom; BV4, diet supplemented with 4 mg BV/kg; BV8, diet supplemented with 8 mg BV/kg; BV12, diet supplemented with 12 mg BV/kg.

Sections from the intestine at different portions (anterior intestine (Fig. 6A and D), middle intestine (Fig. 6E and H), and posterior intestine (Fig. 6I and L)) of different treated groups revealed normal histological structures of columnar epithelial lining, mucosal villi interspersed with goblet cells, lamina propria, submucosal layer, and muscular layer. At which, well-improved intestinal parameters (villus length “VL,” villus width “VW,” absorption surface area “ASA,” and number of goblet cells/villi) at intestinal portions were noticed, particularly at BV8 and BV12. Partially improved intestinal parameters were observed in the BV4 group compared to those in the CONT group. Mucosal villi in the anterior intestine were the longest among intestinal portions. Goblet cells/villi were seen more in middle portions of the intestine in comparison with other intestinal portions. Moreover, there is a gradual increase in numbers of Alcian blue-stained goblet cells that are interspersed within mucosal epithelium from CONT to BV8, with the greatest density observed in BV12 at all different portions of the intestine (Fig. 7). The numerical data of intestinal histomorphology are presented in Table 7.

**Table 7:** Intestinal morphology of *Oreochromis niloticus* following 60 days of dietary bee venom supplementation.

Parameters	CONT	BV4	BV8	BV12	*P*-value
Anterior intestine					
Villous length (µm)	472.33 ± 29.76 ^b^	500.66 ± 2.18 ^ab^	571.66 ± 23.68 ^ab^	680.33 ± 102.02 ^a^	0.102
Villous width (µm)	48.66 ± 7.17 ^b^	73.33 ± 4.80 ^a^	74.66 ± 4.17 ^a^	84.66 ± 6.76 ^a^	0.014
Absorption surface area (mm^2^)	0.022 ± 0.0018 ^c^	0.036 ± 0.0022 ^b^	0.042 ± 0.0015 ^b^	0.056 ± 0.0053 ^a^	0.000
Goblet cells/villi	8.33 ± 0.88 ^c^	15.33 ± 0.88 ^b^	17.33 ± 1.33 ^b^	22.00 ± 1.15 ^a^	< 0.001
Middle intestine					
Villous length (µm)	333.66 ± 11.02 ^b^	343.66 ± 33.94 ^b^	413.66 ± 19.47 ^ab^	442.00 ± 37.44 ^a^	0.063
Villous width (µm)	66.66 ± 6.69	73.66 ± 4.40	68.00 ± 3.51	66.00 ± 3.46	0.662
Absorption surface area (mm ^2^)	0.022 ± 0.0025	0.025 ± 0.0041	0.028 ± 0.0027	0.029 ± 0.0036	0.429
Goblet cells/villi	17.66 ± 1.20 ^b^	39.00 ± 3.60 ^a^	42.00 ± 1.73 ^a^	44.66 ± 1.43 ^a^	< 0.001
Posterior intestine					
Villous length (µm)	424 ± 24.84 ^b^	442.00 ± 17.24 ^ab^	539.66 ± 41.28 ^ab^	568.00 ± 56.32 ^a^	0.070
Villous width (µm)	77.00 ± 4.72 ^b^	74.00 ± 2.08 ^b^	93.00 ± 2.88 ^a^	97.00 ± 7.09 ^a^	0.018
Absorption surface area (mm ^2^)	0.032 ± 0.003 ^b^	0.030 ± 0.001 ^b^	0.050 ± 0.003 ^a^	0.054 ± 0.001 ^a^	< 0.001
Goblet cells/villi	14.00 ± 1.15 ^b^	16.33 ± 0.88 ^b^	18.00 ± 1.52 ^b^	22.00 ± 1.15	0.009

The values are presented as the mean ± SE. Means within the same row carrying different superscript letters are significantly different at *p* < 0.05. CONT, BV4, and BV8: see footnote of Table 4.

### Aeromonas sobria resistance

**Fig. 8:**
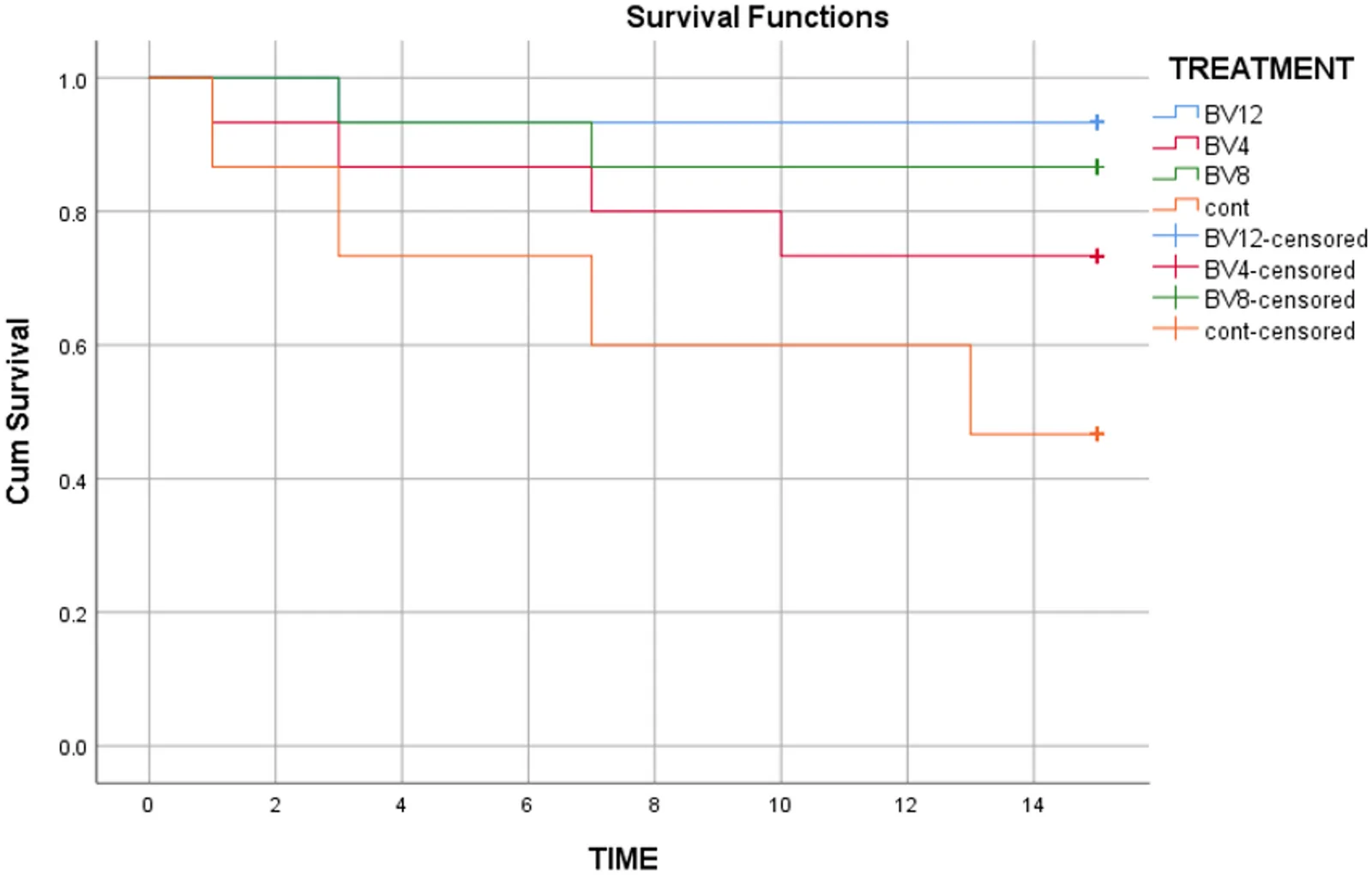
Kaplan–Meier survival curves of *Oreochromis niloticus* fed on bee venom-enriched diets for 60 days and challenged with pathogenic *Aeromonas sobria* for 15 days. Differences among survival curves were evaluated using the log-rank (Mantel–Cox) test (χ² = 10.487, df = 3, *P* = 0.015). CONT, basal diet without bee venom; BV4, diet supplemented with 4 mg BV/kg; BV8, diet supplemented with 8 mg BV/kg; BV12, diet supplemented with 12 mg BV/kg.

The *A. sobria*-challenged fish in the positive control group showed congestion at the base of most of the fins, especially the pectoral and caudal fins along with fin rot. In some cases, recorded scale loss, darkened skin, and eye turbidity. Kaplan–Meier survival analysis demonstrated a significant difference in survival distributions among the challenged groups (log-rank test: χ² = 10.487, df = 3, *P* = 0.015; Fig. 8). By the end of the 15-day observation period, cumulative mortality was 53.33% in the control positive group, compared with 26.66%, 13.33%, and 6.66% in the BV4, BV8, and BV12 groups, respectively. The corresponding 95% confidence intervals of mean survival time were 7.37–13.03, 10.03–14.76, 11.91–15.43, and 12.68–15.72 days for the control, BV4, BV8, and BV12 groups, respectively. Isolation of *A. sobria* was accomplished from freshly deceased fish post-challenge, and these isolates were identified as the challenge bacteria through the identical microbiological identification techniques detailed in the Methods section.

### Economic indicators

The selected economic efficiency indicators of experimental diets are presented in Table 8. Dietary treatments did not significantly affect feed cost per gram of body weight gain (*P* = 0.458). The BV12 group showed the highest calculated profit/g of weight gain (0.0839 ± 0.001) and economic efficiency (81.466 ± 2.249), followed by BV8 and BV4.

**Table 8:** Economic efficiency of the different experimental diets enriched with bee venom.

Treatment	CONT	BV4	BV8	BV12	*P*-Value
Feed $/g BWG	0.00105 ± 0.001	0. 00100 ± 0.001	0. 00101 ± 0.001	0.00103 ± 0.001	0.458
Profit/g weight gain $	0.0589 ± 0.002^d^	0.0724 ± 0.0001^c^	0.0785 ± 0.001^b^	0.0839 ± 0.001^a^	< 0.001
Economic efficiency	56.167 ± 3.095^c^	72.288 ± 2.269^b^	77.821 ± 1.535^ab^	81.466 ± 2.249^a^	< 0.001

The values are presented as the mean ± SE. Means within the same row carrying different superscript letters are significantly different at *p* < 0.05. BWG: body weight gain. $/g diet = 0.0006 for control diet; $0.00064 for BV4 (group fed bee venom 4 mg/kg diet); $0.00068 for BV8 (group fed bee venom 8 mg/kg diet); $0.00072 for BV12 (group fed bee venom 12 mg/kg diet).

## Discussion

Bee venom is one of the natural compounds with numerous benefits, and some recent studies have focused on its properties. However, there is a scarcity of studies investigating the appropriate dosage of bee venom for use as a feed additive for Nile tilapia. Therefore, this study focused on determining the optimum level of inclusion of bee venom in the Nile tilapia diet and its impact on growth performance, immunity, disease resistance, and economic efficiency.

Bee venom is considered a complex biological product due to its numerous proteins, peptides, and enzymes^14,22,51^; it, however, contains several small molecules, which can be characterized using chromatographic methods such as GC-MS. Several types of active metabolites can be found in bee venom; fatty acids, fatty acid esters, long-chain alcohols, phenols, biogenic amines, hydrocarbons, and heterocycles can be regarded as some of the main active compounds of bee venom^52,53^. Active components of bee venom apart from protein are receiving more attention because they may be the reason for the product’s biological activity. It was proven that the mixture of substances that form bee venom produces anti-inflammatory, antibacterial, antioxidant, antiviral, and immunomodulatory activity^21,24,25^. Among all the compounds that were identified in the present study, the highest abundance was recorded for E-11-Tetradecenol trimethylsilyl ether (16.45%), followed by trimethylsilyl (9E)-octadecenoate (11.42%) and palmitic acid TMS derivative (7.87%). Fatty acid derivatives are abundant in bee venom, and this can play an important role in explaining its anti-bacterial, anti-inflammatory, and membrane effects, as lipids do. These results are consistent with the ones found by some researchers that have conducted GC-MS to study the active elements in bee venom and have investigated its toxic and curative effects on the performances of Nile tilapia^12,26^. In the following section, the dose-dependent effect of dietary bee venom on various performance parameters is discussed.

The growth indices are a beneficial indicator used for assessing the impacts of dietary supplements and feed additives on fish performance. A remarkable difference in growth performance was detected in BV-treated groups compared to the CONT group, especially BV12 and BV8. Dietary BV supplementation at 4.2 and 5.8 mg/kg promoted the growth performance of thin-lip mullet throughout the 60-day feeding trial^54^. Similarly, growth indices of red tilapia (*O. mossambicus* x *O. niloticus*) were improved with diet nano-chitosan bee venom complexes (BV-CSNPs) for 70 days in a dose-response pattern with the highest doses at 0.2 and 0.3 mg/kg of diet^27^. In other studies, bee venom was compared with other additives, and bee venom was proven to be the most efficient substance for enhancing the growth rate in Nile tilapia compared to the other additives (liposomal vitamin C and coenzyme Q10) with a diet dose of 4 mg/kg for 60 days of feeding^12^. The enhancement of growth performance in bee venom groups could be related to the improvement in digestion by increasing the activity of digestive enzymes^54^ as well as the improvement in intestinal morphology, resulting in enhancement in nutrient absorption and enhancement of immune-oxidative status^12,54^. The present study shows that the simultaneous enhancement of digestive enzyme activities and the improvement of intestinal morphological parameters provide a plausible link between enhanced digestive function, nutrient absorption, and improved growth performance in the BV8 and BV12 groups. These findings are, however, associations and do not establish a direct causal mechanism. However, no significant differences in juvenile *O. niloticus* growth were found 14 days post-intramuscular injection at varying doses of 3, 6, 9, and 12 mg/kg fish weight^26^. Differences between these results and the reported ones could be related to the method of administration, where bee venom was orally supplemented in this study while intramuscular injection was conducted in the latter study. The routes of administration can impact bioavailability and metabolic pathways and thus the time exposed to the metabolites from bee venom. Also, the fish stage and the ambient conditions could influence the different responses between the studies.

Hematological parameters are an important indicator that provides early, sensitive, and cheap information about aquaculture health because they indicate the status of oxygen transportation and immunity. The present study shows an improvement in Hgb, Hct, MCV, WBCs, and differential leucocytic counts in BV12 and BV8 in comparison to the control group. To the best of our knowledge, no studies have investigated the effects of dietary bee venom on hematological parameters of fish. But two studies have evaluated the effectiveness of bee venom administered either in nanochitosan encapsulation form (BV-CSNPs) incorporated into diet or via intramuscular injections on *O. niloticus*^26,27^. After 14 days of bee venom intramuscular administration, Ht, Hb, and RBCs were unaffected, but an elevation in white blood cells was recorded^26^. Oral supplementation of diet with BV-CSNPs for 70 days caused improved hematological parameters, particularly Hgb, RBCs, PCV, MCH, MCV, and MCHC, especially in the dose of 0.3 mg/kg of diet^27^. Enhancement of these blood parameters might be attributed to the improved functional state of the hematopoietic organs in teleost fish. Bee venom could have immune-enhancing, antioxidant, and anti-inflammatory effects, enhance blood cell production and protect blood cells from oxidative damage^20,55^.

Biochemical parameters are regarded as important indicators of fish physiology, metabolism, and health status^56^. From these parameters, the evaluation of dietary treatment effects on liver and kidney function indices is derived. In this study the ALT, AST, or urea was not influenced by dietary BV supplementation, but only the creatinine was markedly elevated in both BV12 and BV8 compared to the untreated control group. A comparable finding was observed by El Basuini et al.^26^, in which statistically no significant changes were found in ALT, AST, urea, and creatinine in all the *O. niloticus* groups that were injected with varying doses of BV intramuscularly, at 3, 6, 9, and 12 mg BV/kg, after a duration of 14 days. It also was observed that statistically no significant difference was found in the level of ALT, AST, urea, and creatinine in all thin-lip mullets that were fed on varying levels of BV-supplemented diets (2, 4, 6, and 8 mg BV/kg of diet) for 60 days^54^. The insignificant changes of ALT and AST indicated that the dietary bee venom did not significantly affect the hepatic function under the experimental conditions. However, the marked increase in creatinine in the BV8 and BV12 groups should be interpreted with caution. Creatinine may reflect changes in renal function as well as changes in muscle metabolism^57^. Thus, the present findings cannot distinguish whether the increased creatinine was related to increased muscle metabolism associated with growth or to mild renal stress^58^. To understand the biological significance of this finding, an additional assessment of renal biomarkers and tissue histopathology would be needed. The presence of minimal differences in the concentration of urea could be attributed to the fact that the teleost fishes studied are mainly ammonotelic, in which most of the nitrogenous wastes in the form of ammonia are eliminated through the gills. Hence, urea can be considered the secondary substance involved in the elimination of nitrogen and may be less sensitive to physiological renal disturbance^59,60^.

Enzyme activity levels play an essential role in evaluating digestibility and nutrient utilization efficiency, which greatly affect fish growth performance. In this current work, the high dosage of supplementing bee venom (particularly with 8 and 12 mg/kg) caused a significant effect on enhancing digestive enzyme activity (amylase, lipase, and protease) in the serum (*P* < 0.05). In thin-lip mullet, the 2–8 mg/kg diet supplementation of bee venom for 60 days showed a significant increase in the activity of intestinal enzymes amylase, protease, and lipase^54^. In Nile tilapia the highest effect on digestive enzyme activity (lipase, protease, and amylase) was due to supplementing bee venom in the diet (4 mg/kg) compared with other supplementations (liposomal vitamin C and coenzyme Q10) in the diet^12^. In red tilapia diets with bee venom loaded in nano-chitosan, the highest levels of activities of protease, amylase, and lipase were recorded at a dosage of 0.3 mg/kg, and a significant increase of digestive enzyme activities in all groups was observed^27^. The increase of digestive enzyme activities, fed by bee venom, may be related to the increase in integrity of intestinal tissues (layers, villi, and cells) that resulted in enzyme secretion and nutrient absorption^12,27,54^. Meanwhile, bee venom’s antioxidant and anti-inflammatory effects protect the intestine and liver from oxidative stress, and it is favorable for enzyme-secreting cells^26,27^.

The Biochemical indicators of stress are blood glucose and cortisol. These indicators will express stress-related physiological changes in the fish’s body after dietary supplementation. The current study concludes that blood glucose and cortisol levels were similar between control groups and those fed a bee venom-supplemented diet, indicating the doses did not cause a stress response or disrupt metabolic or physiological homeostasis in the fish. Contrary to this finding, the blood glucose level decreased with increasing dose levels of BV-CSNPs in El Basuini et al.^54^ experiment. From a recent study on Nile tilapia, El Basuini et al.^26^ proved that the levels of cortisol and blood glucose were significantly lower than the control when bee venom was administered by IM injection to Nile tilapia. Although the responses appear to be in different directions, the overall meaning of the results is not contradictory. No significant change was observed in this study, and in conjunction with the observed decrease in glucose and cortisol in the literature above, indicating that bee venom supplementation does not have a detrimental effect on the homeostatic balance or stimulation of response in Nile tilapia.

In addition to the determination of indicators from the hematological and biochemical analysis of blood, the estimation of antioxidant capacity may be a useful marker of the physiological state in fish. The study revealed that the antioxidant capacity of the BV-supplemented groups was significantly increased with a marked reduction of MDA, especially in BV12 and BV8 groups. These observed data are also in agreement with studies that have investigated the thin-lip mullet in El Basuini et al.^54^ and Nile tilapia in Teiba et al.^12^ and Eissa et al.^27^. However, El Basuini et al.^26^ stated time-dependent effects on the enzymatic activity of antioxidant enzymes (SOD, CAT, and GPx) in the Nile tilapia’s liver with maximum activity on the 4th and 6th days post-injection.

Apart from antioxidant activity enhancement, dietary bee venom significantly improves the immune parameters (nitric oxide, lysozyme activity, and IgM) and immune gene expression (*IL-1β*,* IL10*, and TNF-α) in the Nile Tilapia, particularly the BV12 and BV8 groups in this present study. The high levels of serum lysozyme activity, antibacterial and burst activity of Nile tilapia fed with a bee venom-supplemented diet (4 mg/kg, 60 days) were significantly greater than other diet additives^12^. The IgM, lysozyme, and bactericidal activity levels of serum and skin mucus of Nile Tilapia increased significantly at the intermediate doses (6–9 mg/kg) after BV intramuscular injection (14 days) and failed to enhance the immune parameters at the low and high doses (3 and 12 mg/kg)^26^. The serum lysozyme activity, bactericidal potency, and nitroblue tetrazolium levels in Thin-lip mullet were positively influenced in BV groups without clear variation among them^54^. *IL-8* and *IL-1β* levels increased as the amount of BV-CSNP supplementation went up, especially with BV-CSNPs 0.3^27^. The immune response benefit observed may be due to the bioactive substances in bee venom such as peptides, enzymes, fatty acids and derivatives, phenolics, long-chain alcohols, heterocyclic compounds, and triterpenoids^13–15,22,24,25,51,52^. These compounds can affect antioxidant and immune-related processes in general. This may account for the simultaneous changes in antioxidant capacity, innate immune parameters, and immune-related gene expression. The present study, however, did not determine the specific contribution of individual venom constituents to these responses.

In the present study, the dose-dependent protective effect of dietary bee venom supplementation on Nile tilapia challenged with bacteria *A. sobria* was clear evidence. Dietary bee venom supplementation at 8–12 mg/kg of diet for 60 d significantly improves disease resistance to this bacterium. The improved survival rates correspond to the remarkable increase in the nitric oxide and lysozyme activities and IgM levels of supplemented groups, as well as the up-regulated expression of *IL-1β*, *TNF-α*, and *IL-10* gene expression in the spleen. Together, these responses may provide for increased immune readiness and increased ability to cope with the bacterial invasion. Nonetheless, as bacterial load and the extent of damage done to the tissues after infection were not measured, it is unclear to what degree these immune responses hindered bacterial growth or tissue damage. Also, the observation agreed with the finding reported by Eissa et al.^27^ on the enhancing effect of BV-CSNPs on the overall health status and challenge of Nile tilapia against *A. hydrophila*. The biological activity of bee venom might be related to the synergistic effects of its numerous components, some of which are the small-molecule compounds identified by GC-MS analysis. While bee venom peptides such as melittin have been reported to possess antibacterial and immunomodulatory activities^15,21,22^, these peptides were not quantified in the present bee venom preparation. Their specific contributions to the observed responses remain speculative and need to be directly characterized in future studies.

In this study, the microscopic structure of the intestinal tract (which serves as the most important absorption site and one of the most significant mucosal immune barriers) did not show any deleterious signs in all tested groups. The morphology of the intestinal villus was not altered in all groups but was more pronounced in the BV-fed group. Villus height and density, epithelial layer integrity, and goblet cell occurrence and distribution were similar in comparison with the control or slightly higher in BV-treated groups. This is in agreement with the detected increasing activities of amylase, lipase, and protease and marked increasing growth in the BV8 and BV12 groups. In the present 60-day trial, under the conditions used, no obvious histopathological changes in the intestine were observed in fish fed up to 12 mg/kg BV. Likewise, the groups treated with BV-CSNPs showed better structures of the intestinal layers, especially the mucosal villi^27^. In addition, intestinal microscopic structure in Nile tilapia^12^ and thin-lip mullet^54^ in the BV-added group was normal. The mucosa, villi, and wall were intact; the villous area was enlarged, and goblet cells were more enhanced in the treated groups than in the CONT.

The economic efficiency indicators calculated in the present study indicated that dietary BV supplementation did not significantly affect feed cost per gram of body weight gain (*P* = 0.458), while the calculated profit per gram of weight gain and economic efficiency were numerically higher in the BV12 group. However, these findings should be interpreted within the scope of the economic indicators used in the present study and should not be considered a comprehensive assessment of the commercial economic feasibility of BV supplementation. Commercial-scale evaluation would additionally require consideration of bee venom production and supply, processing and standardization, transportation, feed manufacturing, and market-scale costs.

From a practical perspective, the use of bee venom as a feed additive in commercial aquaculture requires further evaluation. The availability of bee venom may be associated with the use of productive bee strains, such as the hybridized Carniolan bees used in the present study. However, the study did not assess the sustainable production capacity of bee venom or the feasibility of meeting the needs of commercial aquaculture. Bee venom is widely used in the pharmaceutical and biomedical fields, and large-scale application of bee venom in aquaculture would require careful consideration of availability, production and processing costs, batch-to-batch consistency, storage, transportation, and incorporation into commercial feeds. Scalability of venom collection and the cost of providing the required dietary inclusion levels at farm scale are also yet to be determined. Thus, while the current findings demonstrate beneficial biological responses under experimental conditions, they should be viewed as proof-of-concept data, not proof-of-commercial applicability. Before commercial application can be considered, further studies assessing sustainable venom production, large-scale feed incorporation, long-term safety, and comprehensive economic feasibility at farm scale should be performed.

Some aspects of the present study deserve further investigation. Major components of raw bee venom, such as melittin, apamin, and phospholipase A2, were not characterized by GC-MS analysis. Furthermore, the limited range of dietary doses and 60-day feeding period do not allow definitive conclusions about the entire dose-response relationship or long-term safety. Further investigations using additional renal biomarkers and histopathology should be performed to confirm the high serum creatinine levels of 8 and 12 mg/kg. Finally, although survival in the challenge test was improved, bacterial load and post-challenge tissue histopathology were not evaluated. Future studies should investigate these aspects to better understand the bioactive constituents, safety, and mechanisms of dietary supplementation with bee venom.

## Conclusion

Finally, dietary administration of crude bee venom at 8 and 12 mg/kg significantly enhanced growth performance, digestive enzyme activity, hematological and antioxidant properties, immune response, cytokine gene expression, and disease resistance to *Aeromonas sobria* of Nile tilapia. No clearly observed harmful effects were detected under the experimental conditions of the present study. Considering the results obtained in this experimental condition, dietary bee venom (8–12 mg/kg) may have the potential as a natural feed additive in Nile tilapia. Nevertheless, its application in sustainable commercial aquaculture needs to be validated under farm-scale conditions.

## Data Availability

All data generated or analyzed during this study are included in this article.
